# Getting Up to Date with What Works: A Systematic Review on the Effectiveness and Safety of Task Sharing of Modern Methods in Family Planning Services

**DOI:** 10.1155/2023/8735563

**Published:** 2023-02-07

**Authors:** Tieba Millogo, Eunice Chomi, Seni Kouanda, Moazzam Ali

**Affiliations:** ^1^African Institute of Public Health, 12 P.O. Box. 199 Ouagadougou, Burkina Faso; ^2^Research Institute for Health Sciences, P.O. Box 7192 Ouagadougou, Burkina Faso; ^3^World Health Organization, Avenue Appia 20, Geneva 27, CH-1211, Switzerland

## Abstract

**Objective:**

This systematic review was conducted to provide up-to-date evidence on the safety and effectiveness of task sharing in the delivery of modern contraceptives. *Study Design*. The review followed the Preferred Reporting Items for Systematic reviews and Meta-analyses (PRISMA) guidelines. We searched Medline, Embase, Cochrane CENTRAL, and Google Scholar for peer-reviewed studies that reported on effectiveness and/or safety outcomes of task sharing of any modern contraceptive method. Only Cochrane Effective Practice of Organizations of Care (EPOC) study designs were eligible, and quality assessment of the evidence was performed using the Cochrane risk of bias (RoB) tools. Meta-analyses, where possible, were carried out using Stata, and certainty of the evidence for outcomes was assessed using the Grading of Recommendations Assessment, Development, and Evaluation tool (GRADE).

**Results:**

Six studies met the inclusion criteria: five reported on self-injection of subcutaneous depot medroxyprogesterone acetate (DMPA-SC) compared to administered by trained health providers; and one assessed tubal ligation performed by associate clinicians compared to advanced-level associate clinicians. Self-injection improved contraceptive continuation, with no increase in unintended pregnancy and no difference in side effects compared to provider administered. In tubal ligation, the rate of adverse events, time to complete procedure, and participant satisfaction were similar among associate clinicians and advanced clinicians.

**Conclusion:**

The evidence suggests that self-injection of DMPA-SC and tubal ligation performed by associate clinicians are safe and effective. These findings should be complemented with the evidence on the feasibility and acceptability of task sharing of these methods. The review protocol was registered with PROSPERO CRD42021283336.

## 1. Introduction

The global shortage of health workforce is increasingly being recognized as a major impediment to the coverage of essential services [1] and constitutes one of the drawbacks to achieving universal health coverage (UHC). The current unmet needs for health workers that disproportionately affect low- and middle-income countries (LMICs) are anticipated to remain unaddressed for the next decade [2]. It is projected that by 2030, there will be a shortage of 15 million health workers globally [3]. In addition, inequities in the distribution of the existing health workforce within countries, with important rural versus urban differentials in both number and qualification, are worsening the coverage of services in the most deprived, yet high demand areas [4]. Family planning services are highly affected by both workforce shortages and inequitable distribution, creating a vicious circle of suboptimal coverage of services fueling back a growing demand for services [5–7]. Unravelling this vicious circle will take a dramatic increase in both numbers and qualifications of healthcare workers that is unlikely to happen in the short and midterm.

Task sharing of contraceptive delivery to low- and midlevel health cadres of health workers has been recognized as a promising strategy to address health worker shortages and limited access to modern contraceptive methods [8–10]. Task sharing involves the training of mid- and low-level cadres of health workers to safely and effectively deliver services that were performed by higher level cadres, as a mean of expanding access to health services with a limited health workforce [9, 11]. Low- and midlevel cadres of health workers for the provision of a specific contraceptive method refer to cadres of health workers whose qualification is below that of the cadres of health workers tasked with the duty of providing this method per prevailing guidelines, providing that the targeted method is not considered outside their typical scope of practice and competencies. However, uncertainties remain [8] regarding which methods can be safely and effectively delivered by the different cadres of health workers given their training backgrounds.

The 2012 World Health Organization (WHO) document “Optimizing health worker roles to improve access to key maternal and newborn health interventions through task shifting” highlighted specific areas where knowledge gaps were noted [9]. These areas included evidence on the safety and effectiveness of task sharing in (1) delivery of tubal ligation by nurses and midwives; (2) delivery of vasectomy by auxiliary nurses, auxiliary nurse-midwives, nurses, and midwives; (3) delivery of injectable contraceptives using a compact, prefilled autodisabled device (CPAD) such as Uniject™ by lay health workers (LHWs), auxiliary nurses, and auxiliary nurse-midwives; (4) insertion and removal of intrauterine derives (IUDs) by auxiliary nurses; and (5) the delivery of contraceptive implants by lay health workers. The WHO defines LHW as “Any health worker who performs functions related to healthcare delivery; was trained in some way in the context of the intervention; but has received no formal professional or paraprofessional certificate or tertiary education degree.”

In addition, the advent of subcutaneous depot medroxyprogesterone acetate (DMPA-SC) has introduced the possibility of self-injection. WHO guidelines on self-care interventions for health acknowledge that it is another important component of task sharing, noting that “Women themselves have a role to play in managing their own health and this constitutes another important component of task sharing within health systems” [10].

The purpose of this review was to review the latest evidence since the publication of the “WHO guidelines on task sharing” [9] for all modern contraceptive methods including the gaps noted above to update it.

## 2. Material and Methods

The review was conducted following the Preferred Reporting Items for Systematic reviews and Meta-analyses (PRISMA) guidelines [11]. Two review authors (TM and EC) performed the database search, screening, extraction, and quality assessment independently and compared/cross-validated the results. TM performed the analysis. A third review author was available for consultation in the event of a disagreement between the first two that could not be settled through discussions. The review protocol was registered with PROSPERO (CRD42021283336).

### 2.1. Criteria for the Selection of Studies

#### 2.1.1. Types of Studies

Based on the Cochrane Effective Practice of Organizations of Care (EPOC) group criteria for study designs eligible for evaluating the effects of healthcare interventions, this review included quantitative, peer reviewed studies [12]. We included Randomized Controlled Trials (RCTs), Nonrandomized Controlled Trials (NRCTs), controlled before-after study designs, interrupted time series, repeated measures study designs, and Cluster Randomized Controlled Trials (CRCTs).

#### 2.1.2. Types of Interventions

The types of contraceptive interventions included in the search were as follows: (i) the provision of oral contraceptives and condoms; (ii) the provision of injectable contraceptives using standard syringe or an autodisabled, prefilled injection device such as Uniject™, DMPA-SC; (iii) insertion and removal of contraceptive subdermal implants; (iv) insertion and removal of IUDs; (v) tubal ligation; and (vi) vasectomy. We searched studies in which the interventions were self-delivered or delivered by Patent Proprietary Medicine (PPM) drug vendors, pharmacists and or pharmacy workers, lay health workers, auxiliary nurses, auxiliary nurse-midwives, nurses, midwives, associate clinicians, and advanced associate clinicians (for search terms and search strategy, see Appendix A). The delivery of the contraceptive methods was considered a form of task sharing in studies where it was carried out by cadres of health workers that were less qualified than the cadres of health workers normally tasked with the delivery of these methods per prevailing guidelines in the study setting. We included self-injection as a task-sharing strategy in line with the WHO guidance that spans task sharing to “entrusting certain aspects of healthcare to individuals to assess and manage their own care, including family planning” [9, 10].

#### 2.1.3. Types of Comparisons

We included studies that compared the intervention to standard care, here defined as the performance of the intervention by cadres of healthcare workers that are normally assigned this role per health policies and regulations of the study setting. The standard care encompasses situations where the cadres of healthcare workers involved in the comparison group are well defined and situations where this was not precisely defined but assumed to be the standard approach for delivering the contraceptive intervention in the setting being considered.

#### 2.1.4. Populations and Settings

Adult men and women in reproductive age groups were eligible for this review. We included women in reproductive age who received the intervention antenatally, in postpartum periods, or during postabortion care. No setting-related restriction was applied. Participants recruited from both community and health facility settings were eligible for the review, and we included studies conducted in rural, urban, and periurban areas.

#### 2.1.5. Types of Outcomes

We included studies that assessed the safety and effectiveness of task-sharing contraceptive interventions. Effectiveness was defined as the extent to which task sharing for the delivery of a contraceptive intervention was successful in achieving the desired outcomes (benefits of contraceptive use). Examples of effectiveness outcomes were anticipated to be contraceptive continuation rates and unintended pregnancy rates. Studies reporting on population-level effectiveness outcomes such as contraceptive coverage were also eligible. Safety was defined as the extent to which an intervention was delivered safely by a particular cadre of health workers. Safety outcomes included adverse events and side effects such as infections, pain swelling, and needlestick injuries. The safety and effectiveness outcomes considered were anticipated to vary according to the contraceptive methods (review of outcomes, see Appendix B). User satisfaction was considered a secondary outcome in this review and included only for those studies that reported on satisfaction in addition to other effectiveness and/or safety outcomes.

#### 2.1.6. Studies Excluded

We excluded studies that did not report outcomes relevant to this review, such as contraceptive or performance knowledge of providers and/or recipients; knowledge, attitudes, and intentions of recipients; and adherence to standards in the provision of the methods by cadres of health workers. Further exclusion criteria were (i) studies reporting on promotional activities or task-sharing interventions that did not test a specific contraceptive method and where the measured outcomes could not be directly linked to a specific contraceptive method and (ii) studies reporting on outcomes of contraceptive counseling by other cadres of healthcare workers aside of self-care, because we considered this to be known as safe and effective [9].

### 2.2. Search Methods

We searched major global electronic databases (Medline, Embase, POPLINE, Cochrane CENTRAL, and Google Scholar). Using the PICO (Population, Interventions, Comparison, Outcomes) approach (for PICO questions, see Appendix C) [13], we searched the literature based on three concepts: population (cadres of health workers), intervention (contraceptive methods), and outcomes (safety, effectiveness). In Embase, CENTRAL, and Medline databases, search terms related to the three concepts included controlled vocabulary (medical subject headings (MeSH) for Medline and CENTRAL and Emtree for Embase) and free-text words. The Boolean operator “OR” was used with synonyms within each concept; then, the search results for the different concepts were combined with the “AND” operator. The search terms and search strategy (for search terms and search strategy, see Appendix A) were developed by TM with input from a WHO technical lead in database search and an experienced WHO librarian. Some of the search terms were used in truncated formats to maximize the yield of the search. We used restriction features available in the different databases to select studies pertaining to 2013 onwards. We used key terms and free texts to search Google Scholar. Reference lists of included studies were hand searched for additional studies. Two review authors (TM and EC) independently ran the search of the databases. The first search was run on 5^th^, 6^th^, and 7^th^ February 2021. We reran the search in all databases on 21^st^ November 2021 to update any new evidence available. The papers found in the databases were exported into a reference management software (Zotero) for further assessment.

### 2.3. Data Screening, Collection, and Analysis

Duplicate studies were removed in Zotero. One review author screened the titles of the articles to exclude obviously nonrelevant papers. Then, two review authors assessed the abstracts of the remaining papers and retrieved the full-text versions of relevant studies. The full texts were further assessed independently by the review authors to determine eligibility. Any discrepancies between review authors at either stage of the selection of studies was resolved through discussion to reach a consensus. We involved a third review author if the first two authors failed to reach consensus on a study. Data were extracted using a standardized extraction form developed and agreed upon by the review authors.

Quality of included studies was assessed using the risk of bias (RoB) tools developed by Cochrane for RCTs (ROB2) and NRCTs (ROBINS-I) [14]. The review authors independently assessed the RoB for each outcome in included studies and graded the risk from low to critical. Discrepancies were resolved by mutual consent and discussion. The certainty of evidence for primary outcomes was assessed using the GRADE tool [15].

Findings for task sharing to each cadre of healthcare worker and on each contraceptive method were presented separately. Because of the few numbers of studies and diversity in the outcomes reported, a meta-analysis was not possible for all the outcomes except contraceptive continuation rate and overall side effects. Meta-analyses were performed using Stata Software (Stata Corp, College Station, Texas, USA; version 15.1). Narrative synthesis was performed where meta-analyses were found not relevant or practically unfeasible. We presented the results individually and as risk ratios with 95% CI. In the meta-analyses, the choice between fixed and random effects models (with inverse variance method) to compute the summary effect measures was determined by statistical heterogeneity, measured by the percentage of between-study heterogeneity that is attributable to variability in the true treatment effect, rather than sampling variation (*I*^2^ statistic) [16]. We recalculated effect measures for individual studies using actual numbers of events when possible and needed to have standardized outcome definitions across studies.

## 3. Results

### 3.1. Results of the Search

**Figure 1:**
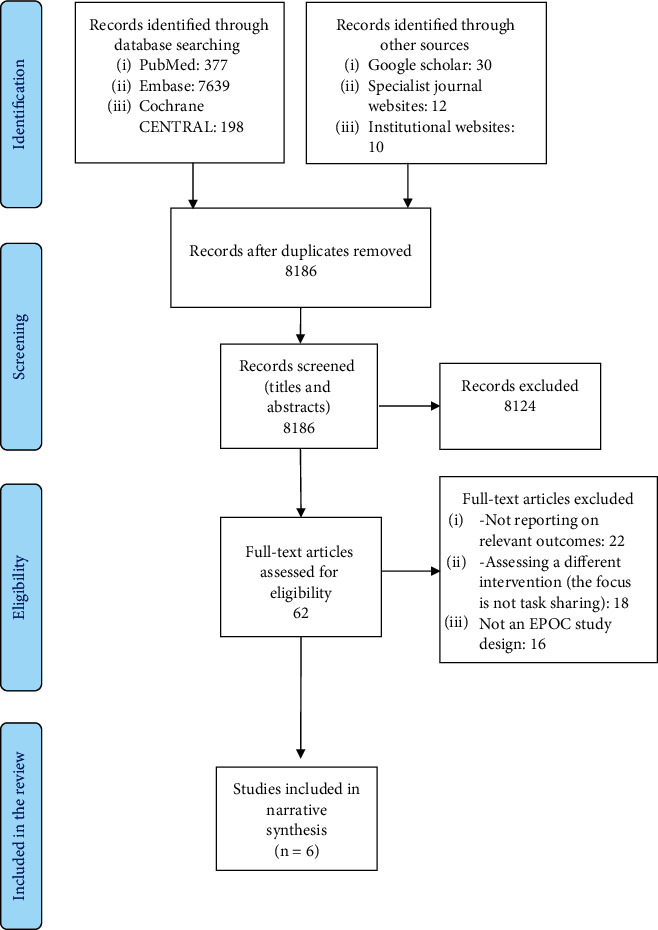
PRISMA flowchart.

The database search yielded 8266 records in total. After screening the titles and abstracts of the records, full texts of 62 studies were retrieved for further assessment. Finally, 6 studies (4 RCTs and 2 NRCTs study designs) met the inclusion criteria and were included in the review (see Figure 1). These studies assessed the following: (i) self-injection of DMPA-SC compared to usual care, i.e., facility-based administration (subcutaneous/intramuscular DMPA) by qualified healthcare workers (3 RCTs and 2 NRCTs), and (ii) tubal ligation performed by associate clinicians compared to advanced-level associate clinicians (1 RCT). All included studies were conducted at subnational level and took place in health facilities, at the community level or both. The PRISMA flowchart summarizing the screening and selection of final studies for the review is provided in Figure 1.

### 3.2. Dearth of New Evidence on Research Gaps

Beside elaborating on new evidence, this review also points to the lack of evidence on many task-sharing interventions for family planning (FP) services based on ‘WHO guidelines on task sharing' [9, 17]: The identified research gaps included the following:

- Provision of injectable contraceptives by LHWs and pharmacy workers

Due to lack of rigorous research data on the effectiveness and safety of provision of injectable contraceptives by both cadres of health workers, WHO's 2012 recommendations only recommend the provision of injectable contraceptives by LHWs and pharmacy workers in specific circumstances. In the present review, we found six studies reporting on the provision of injectable contraceptives by LHWs [18–23], none on the provision by pharmacy workers. Of the six studies, which report on task-sharing injectable contraceptives to LHWs, four were single-arm studies merely reporting descriptive outcomes without any comparison group [19, 20, 21, 23], one study reported on performance outcome of LHWs [22] (ability to adequately perform injections) and the last one compared community-based provision by LHWs to facility-based provision by a different cadres of health workers including LHWs [18]. These studies did not meet inclusion criteria for this review and could not further contribute to the evidence.

(ii) Implant insertion and removal by LHWs, auxiliary nurses, and auxiliary nurse-midwives

The present review found one study that reported on task sharing of contraceptive implants to LHWs [24]. This was a single-arm study investigating the feasibility of the intervention without effectiveness or safety outcome, and no comparison group was involved.

(iii) Insertion and removal of IUDs by auxiliary nurses

No eligible study reporting on the said outcome was found. The search retrieved nine studies focused on insertion of contraceptive intrauterine devices by other cadres of healthcare providers (nurses, midwives, and advance clinicians) in Sri Lanka, India, Nepal, Bangladesh, Tanzania, Kenya, Malawi, Australia, United Kingdom, and Brazil [25–33]. None of these studies used an EPOC study design.

(iv) Vasectomy by auxiliary nurses, auxiliary nurse-midwives, nurses, and midwives

The search found no study on this intervention.

(v) Tubal ligation by nurses and midwives

We found no study reporting on this intervention. Three studies were found reporting on task sharing of tubal ligation to other cadres of health workers (clinical officers and assistant medical officers) [34–36]. Of these studies, two were single-arm studies reporting descriptive outcomes without any comparison group and were excluded from the review [34, 35].

### 3.3. Summary of the Evidence Found

#### 3.3.1. Characteristics of Included Studies

Of the five studies on self-injection, two compared self-injection of DMPA-SC to facility-based administration of intramuscular depot medroxyprogesterone acetate (DMPA-IM) [37, 38] and three studies compared self-injection of DMPA-SC to facility-based administration of DMPA-SC [39–41]. All the studies reported on 12-month continuation rates, and three studies reported on user's satisfaction. Unintended pregnancy rates were also reported. The safety outcomes reported were injection site reactions (pain, indentation) and side effects.

The study on task sharing for tubal ligation [36] reported on safety, looking at the rate of major adverse events, time to complete procedure, request of a supervisor assistance, and users' satisfaction. Basic characteristics of the included studies are presented in Table 1.

#### 3.3.2. Self-Injection of DMPA-SC

The results of the five studies assessing this intervention suggest the following (see Table 2):

- Contraceptive continuation is higher with self-injection of DMPA-SC compared to facility-based administration. This finding was consistent across all studies, with self-injectors 38% less likely to discontinue at 12 months in the 2 NRCTs (RR: 0.62; 95% CI: 0.47-0.82; *p* = 0.001, moderate certainty evidence) and 44% less likely to discontinue at 12 months in the 3 RCTs (RR: 0.56; 95% CI: 0.37-0.86; *p* < 0.01, moderate certainty evidence). Noteworthy, one study suggests interaction between self-injection and age of the women, where self-injection improved contraceptive continuation among young women aged 18-24 years by 40% compared to 25% among those aged 25 years and above [37]
- There is little or no difference between self-injection and facility-based administration of DMPA-SC in overall side effects experienced by contraceptive users (RR: 0.86; 95% CI: 0.77 to 0.96, low certainty evidence)
- Both RCT and NRCT results suggest there is no or little difference between self-injection and facility-based administration groups in the occurrence of unintended pregnancies (very low certainty evidence)
- There is little or no difference in users' satisfaction between self-injection and facility-based administration groups (moderate certainty evidence)

#### 3.3.3. Tubal Ligation by Associate Clinicians

The study comparing tubal ligation performed by *associate clinicians* (a professional clinician with basic competencies to diagnose and manage common medical, maternal, child health, and surgical conditions. They may also perform minor surgery. The prerequisites and training can be different from country to country. However, associate clinicians are generally trained for 3- to 4-year postsecondary education in established higher education institutions. The clinicians are registered, and their practice is regulated by their national or subnational regulatory authority [9]) and *advanced associate clinicians* (a professional clinician with advanced competencies to diagnose and manage the most common medical, maternal, child health, and surgical conditions, including obstetric and gynecological surgery (e.g., caesarian sections). Advanced-level associate clinicians are generally trained for 4- to 5-year postsecondary education in established higher education institutions and/or 3-year postinitial associate clinician training. The clinicians are registered, and their practice is regulated by their national or subnational regulatory authority [9]) suggests the following (see Table 3):

- There is no difference or a lower rate of adverse events when the tubal ligation is performed by associate clinicians compared to advanced associate clinicians (RR: 0.00050; 95% CI: 0.00007 to 0.00360, moderate certainty evidence; RR: 1.5; 95% CI: 0.3 to 8.9, moderate certainty evidence for major adverse events and overall adverse events, respectively)
- Time to complete the procedure is identical between associate clinicians and advanced associate clinicians with mean time of 26.0 minutes and standard deviation of 1 in each group (mean difference: 0 min; 95% CI: -0.09 to 0.09, high certainty evidence)
- Participant satisfaction is similar when tubal ligation is performed by associate clinicians or advanced associate clinicians (RR: 1.01, 95% CI: 0.88-1.15, moderate certainty evidence). Maximum pain during the procedure is similar when performed by associate clinicians and advanced associate clinicians (mean score difference: 0.01, 95% CI: -0.2 to 0.22, moderate certainty of evidence)
- There is no difference in the need for support with the procedure between associate clinicians and advanced-level associate clinicians: RR: 1.08; 95% CI: 0.47-2.52 (moderate certainty evidence)

## 4. Discussion

The purpose of this review was to synthesize the evidence on effectiveness and safety of task sharing of a wide range of contraceptive methods to strengthen the existing recommendations and to update new ones in the WHO guideline on task sharing for modern methods [9]. The review identified new eligible evidence on two methods: (i) self-injection of subcutaneous injectable contraceptives versus facility-based administration and (ii) provision of tubal ligation by associate clinicians versus advanced-level associate clinicians.

We found no eligible study on task-sharing injectable contraceptives to LHWs, task sharing of IUDs/post-partum intrauterine devices (PPIUDs) and implant insertion, or vasectomy. This dearth of data on some contraceptive interventions is not new. A previous review covering a different time frame also found no study on task sharing of implant insertion [17]. This may be explained by a combination of two factors: insufficient research data in the literature on these methods or available data generated through poor quality research designs that do not qualify or meet standards for the evaluation of the effectiveness of healthcare interventions [12]. Publications found in our search that focused on task sharing of injectable contraceptives to LHWs [19–21, 23], of IUDs/PPIUDs [25, 26, 28–32], and of contraceptive implants [24] did not meet the inclusion criteria to be included in the present review.

The review also found that self-injection significantly improves contraceptive continuation without additional harm. A similar finding was reported by a previous review that did not however grade the certainty of the evidence for each review outcomes [42]. The lower risk of method discontinuation found in our review is an invaluable result [43]. Indeed, injectable contraceptives are highly effective and popular contraception methods in LMIC, yet characterized by significant discontinuation rates affecting their potential to achieve the goal of contraceptive use, i.e., avoiding unintended pregnancies [44, 45]. The results suggest that adopting self-injection of DMPA-SC may not only expand geographic coverage through a new delivery option but will also improve the adherence in utilization without exposing contraceptive users to additional risk of adverse events or complications.

Overall, the certainty of the evidence was low or very low for most of the outcomes reported for self-injection except contraceptive continuation, which was the only stated primary outcome for most of the studies. This suggests the need for robust design in future studies focusing on safety and effectiveness outcomes such as unwanted pregnancies. To the best of our knowledge, our review was the first in going beyond the quality of the evidence for individual studies to systematically assess the quality of the evidence for each reported outcomes across self-injection studies. The findings did not contradict what is already known on self-injection [46], and it also highlighted areas where strong evidence is still needed. Part of these areas is the capacity to adequately manage the waste (storage, destruction, etc.) generated by self-injection.

Self-injection was not included in the previous WHO recommendations; our findings suggest that there is increasing evidence in the literature to consider this option when revising the current recommendations, even though more robust studies from different settings are still needed.

The findings on tubal ligation suggest that associate clinicians are not inferior to advanced associate clinicians in performing tubal ligation. Despite that only one study is included for this review question, the results are important as this was, to the best of our knowledge, the first review where the noninferiority question was addressed using good study design. The previous recommendations by the WHO on the performance of tubal ligation by associate and advanced associate clinicians were based on the consideration that this intervention lies within their typical scope of practice [9]. Our findings support and reinforce this recommendation.

The search for task-sharing studies is challenging because of the varying terminologies for the different cadres of healthcare workers across settings. Therefore, we cannot rule out that our search might have missed some relevant studies. The inconsistencies in defining effectiveness and safety outcomes for the same method across studies also made it challenging, if not impossible to report results in terms of average combined effects of the interventions. Finally, the applicability of the findings to any setting might also depend on the implementation environment (health system, resources, trainings, and sociocultural backgrounds) and could shape the implementation results. Health literacy of contraceptive users, for example, may vary across settings and is important when considering self-injection. Different training schemes for the same terminology used for cadres of healthcare workers may also be the source of differences in competences that may be worth taking into consideration.

## 5. Conclusion

This review reported on new evidence on task sharing in the provision of contraceptive methods. We found no eligible study on task sharing of contraceptive implants, IUDs, and vasectomy. The findings support previous knowledge in the area and stress persistent knowledge gaps that need to be fulfilled. An important and new finding was the safety and effectiveness of self-injection of DMPA-SC. The current WHO recommendations on task sharing for modern contraceptive methods do not include self-injection. Based on the evidence, it will be worth considering inclusion of self-injection and updating the current WHO recommendations.

The results of this review on safety and effectiveness need to be complemented with the available evidence on the feasibility and acceptability of task sharing, including from mixed-methods and qualitative studies. The dearth of knowledge on task sharing must be addressed along with steps to maximize comparability and replicability of results. Future studies should clearly state and define primary and component outcomes when reporting composite outcomes and provide data at each end point. In addition, using robust study designs, conduct studies with pregnancy outcomes (unwanted pregnancy, abortion) as primary outcomes of task sharing and where possible, consider minimizing self-reporting of safety and effectiveness outcomes.

## Figures and Tables

**Table 1:** Basic characteristics of studies included in the review.

1^st^ author, year	Study site	Study design	Study size, population	Interventions	Outcomes reported
Anitra Beasley, [40]	Health facilities and community, New York, USA	RCT	132 women ≥ 18 years	Self-injection compared to facility-based administration of DMPA-SC	12-month contraceptive continuation: 47% and 48% for the self and clinic administration groups (*p* = 0.70). MPA levels in serum: similar in both groups (686.2 ± 318.5 pg/ml *vs*. (695.8 ± 309.6 pg/ml)
Holly Burke, [39]	Health facilities and community, rural Malawi, Africa	RCT	731 women ≥ 18 years	Self-injection compared to facility-based administration of DMPA-SC	12-month contraceptive continuation: 73% in the self-injection group and 45% in the provider-administered group (log-rank *p* < 0 · 0001). Side effects: reported by ten women (20 events) in the self-administered group and 17 women (28 events) in the provider-administered group (*p* = 0.24)
Jane Cover, [37]	Health facilities and community, Uganda, Africa	NRCT	1161 women ≥ 18 years	Self-injection of DMPA-SC compared to facility-based administration of DMPA-IM	12-month contraceptive continuation: 0.81 (95% CI.78–.84) in the DMPA-SC group, 0.65 (95% CI 61–.69) in DMPA-IM group (*p* < 0.05). Side effects, adverse events: greater percentage of reports among the DMPA-IM group, more injection site reactions among the DMPA-SC group
Jane Cover, [38]	Health facilities and community, Senegal, Africa	NRCT	1299 women ≥ 18 years	Self-injection of DMPA-SC compared to facility-based administration of DMPA-IM	12-month contraceptive continuation: 80.2% in the self-injecting group, 70.4% in the provider-administered group (*p* < 0.01). Side effects, adverse events: fewer reports among the self-injection group compared to the provider-administered group
Julia E Kohn, [41]	Health facilities and community, Texas and New Jersey	RCT	401 women ≥ 15 years	Self-injection compared to facility-based administration of DMPA-SC	12-month contraceptive continuation: 69% among self-injectors vs. 54% among the provider-administered group (*p* = 0.05). Side effects: no serious side effects reported. Satisfaction: 87% among self-injectors vs. 92% among the provider-administered group
Mark A Barone, [36]	Health facilities, Tanzania, Africa	RCT		Tubal ligation performed by associate clinicians (CO) versus advanced associate clinicians (AMO)	Rate of major adverse effects: 0% in the CO group, 0.1% in the AMO group (RD-0.1%, 95% CI -0.3%-0.1%). No differences in participant satisfaction between the groups

**Table 2:** Summary of findings on self-injection of DMPA-SC.

Is self-injection of injectable contraceptives safe and effective compared to usual care?						
Patient or population: injectable contraceptive users Setting: Texas and New Jersey (USA), New York (USA), Malawi, Senegal, Uganda (Africa) Intervention: self-injection Comparison: usual care						
Outcomes	Anticipated absolute effects^∗^ (95% CI)		Relative effect (95% CI)	No. of participants (studies)	Certainty of the evidence (GRADE)	Comments
	Risk with usual care	Risk with self-injection				
Contraceptive discontinuation (12 months) (discontinuation)	51 per 100	29 per 100 (19 to 44)	RR 0.56 (0.37 to 0.86)	1142 (3 RCTs)	⨁⨁⨁◯ Moderate^a^	
Contraceptive discontinuation (12 months) (discontinuation)	31 per 100	19 per 100 (14 to 25)	RR 0.62 (0.47 to 0.82)	2398 (2 observational studies)	⨁⨁⨁◯ Moderate^b,c^	
Overall side effects after 3rd injection (side effects) assessed with: self-reporting	23 per 100	19 per 100 (17 to 22)	RR 0.86 (0.77 to 0.96)	2052 (2 observational studies)	⨁⨁◯◯ Low^d^	
Nondesired pregnancies (pregnancy rate) assessed with: urine pregnancy tests (1 study), self-reporting (1 study)	Ten nondesired pregnancies occurred: 6 in the control group and 3 in the intervention group. Results suggest no or little difference between control and intervention			1132 (2 RCTs)	⨁◯◯◯ Very low^e,f^	
Nondesired pregnancies (pregnancy rate) assessed with: not stated	Six unplanned pregnancies were reported, half in each arm. Results suggest no or little difference between control and intervention			2398 (2 observational studies)	⨁◯◯◯ Very low^b,f^	
^∗^The risk in the intervention group (and its 95% confidence interval) is based on the assumed risk in the comparison group and the relative effect of the intervention (and its 95% CI). CI: confidence interval; RR: risk ratio						
GRADE Working Group grades of evidence High certainty: We are very confident that the true effect lies close to that of the estimate of the effect Moderate certainty: We are moderately confident in the effect estimate: The true effect is likely to be close to the estimate of the effect, but there is a possibility that it is substantially different Low certainty: Our confidence in the effect estimate is limited: The true effect may be substantially different from the estimate of the effect Very low certainty: We have very little confidence in the effect estimate: The true effect is likely to be substantially different from the estimate of effect						

^a^The proportion of the variability in effect estimates that is due to true heterogeneity rather than chance is important *p* = 0.015. ^b^Downgraded because of concerns over residual confounding. ^c^The comparison group here was provider administration of DMPA-IM instead of DMPA-SC. We did not downgrade the evidence because DMPA-IM and DMPA-SC are considered very similar aside the route of administration. ^d^Risk of bias assessment was “serious” for both studies on this outcome per ROBINS-I. ^e^One in 2 studies reported important missing data (>15%) in the outcome with clear imbalance between intervention and control groups. ^f^Because of the few numbers of events, effect measures were not computed in individual studies.

**Table 3:** Summary of findings on tubal ligation by associate clinicians.

Is tubal ligation performed by associate clinicians compared to tubal ligation performed by advanced-level associate clinicians safe and effective?						
Patient or population: minilaparotomy clients Setting: Tanzania, Africa Intervention: tubal ligation performed by associate clinicians Comparison: tubal ligation performed by advanced-level associate clinicians						
Outcomes	Anticipated absolute effects^∗^ (95% CI)		Relative effect (95% CI)	No. of participants (studies)	Certainty of the evidence (GRADE)	Comments
	Risk with tubal ligation performed by advanced-level associate clinicians	Risk with tubal ligation performed by associate clinicians				
Overall major adverse events (42-day postsurgery) (MAEs)	0 per 1000	0 per 1000 (0 to 0)	RR 0.00050 (0.00007 to 0.00360)	1962 (1 RCT)	⨁⨁⨁◯ Moderate^a^	
Major and minor adverse events (7-day postsurgery)	2 per 1000	3 per 1000 (1 to 18)	RR 1.5 (0.3 to 8.9)	976 (1 RCT)	⨁⨁⨁◯ Moderate^a^	
Time to complete procedure (duration)	The mean time to complete procedure was 26.0 mn	0 mn (0.09 lower to 0.09 higher)	—	1962 (1 RCT)	⨁⨁⨁⨁ High	
Request supervisor assistance with procedure	15 per 1000	17 per 1000 (7 to 39)	RR 1.08 (0.47 to 2.52)	1962 (1 RCT)	⨁⨁⨁◯ Moderate^a^	
Participant satisfaction	861 per 1000	869 per 1000 (757 to 990)	RR 1.01 (0.88 to 1.15)	1945 (1 RCT)	⨁⨁⨁◯ Moderate^b^	
Maximum pain during procedure (pain)	The mean maximum pain during procedure was 4.12	Mean 0.01 higher (0.2 lower to 0.22 higher)	—	1962 (1 RCT)	⨁⨁⨁◯ Moderate^b^	
^∗^The risk in the intervention group (and its 95% confidence interval) is based on the assumed risk in the comparison group and the relative effect of the intervention (and its 95% CI). CI: confidence interval; RR: risk ratio						
GRADE Working Group grades of evidence High certainty: We are very confident that the true effect lies close to that of the estimate of the effect Moderate certainty: We are moderately confident in the effect estimate: The true effect is likely to be close to the estimate of the effect, but there is a possibility that it is substantially different Low certainty: Our confidence in the effect estimate is limited: The true effect may be substantially different from the estimate of the effect Very low certainty: We have very little confidence in the effect estimate: The true effect is likely to be substantially different from the estimate of effect						

^a^Downgraded due to the very small number of events. ^b^Downgraded over concerns on measurement bias (subjective outcome and no blinding).

## Data Availability

We did not use any raw database, and the papers reviewed are indexed in the reference section.

## Supplementary Materials

Supplementary 1 Appendix A: search terms and search strategy.

Supplementary 2 Appendix B: review outcomes by contraceptive methods.

Supplementary 3 Appendix C: for PICO questions.
