# Supplementary material for: Getting Up to Date with What Works: A Systematic Review on the Effectiveness and Safety of Task Sharing of Modern Methods in Family Planning Services
Source: Biomed Res Int. 2023 Feb 7;2023:8735563. doi: 10.1155/2023/8735563 (PMC9936454; doi:10.1155/2023/8735563)
Supplement: Supplementary 1 — Appendix A: search terms and search strategy. [file 8735563.f1.pdf]

PubMed (5th , November 2020 and 5th February 2021)

**40**	#28 AND #37 AND #27	348
**39**	#28 AND #37 AND #27	348
**38**	#28 AND #37 AND #27	348
**37**	#36 OR #29	26,385
**36**	#33 AND #29	6,96
**35**	#28 AND #34 AND #27	4,573
**34**	#33 OR #29	127,42
**33**	Counseling [MeSH] OR Contraceptive Effect [MeSH] OR Informed choice counseling [tw] OR Counselling [tw] OR counselling strategies [tw] OR informed choice counselling [tw] OR structured counselling [tw] OR video counselling [tw] OR face-to-face counselling [tw] OR leaflet-based counselling [tw] OR telephone-based counselling [tw] OR individual counselling [tw] OR group counselling, husband counselling [tw] OR couples counselling [tw]	107,983
**32**	#1 AND #28 AND #29 AND #27 AND #26	27
**31**	#28 AND #29 AND #27	348
**30**	#1 AND #28 AND #29 AND #27	317
**29**	#15 OR #17 OR #19 OR #21 OR #23 OR #24	26,384
**28**	#2 OR #3 OR #5 OR #7 OR #8 OR #9 OR #10 OR #11 OR #13	972,095
**27**	2012 [DP]: 2020 [DP]	10,085,213
**26**	safety management [MeSH] OR Patient satisfaction [MeSH] OR Personal satisfaction [MeSH] OR cost-benefit analysis [MeSH]	210,043
**24**	Sterilization, Tubal [MeSH] OR Aldridge Procedure [tw] OR Cooke Method [tw] OR Cornual Coagulation [tw] OR Fimbriectomy [tw] OR Irving Method [tw] OR Tubal Occlusion, Nonchemical [tw] OR Ligation, Tubal [tw] OR Madlener Method [tw] OR Pomeroy Method [tw] OR Tubal Occlusion [tw] OR Tubal Occlusion, Chemical [tw] OR Uchida Method [tw] OR Kroener Fimbriectomy [tw]	5,08
**23**	Vasectomy [MeSH] OR Vas Ligation [tw] OR Vas Occlusion [tw] OR Vas Occlusion, Nonchemical [tw] Vasectomies [tw] OR vasectomy [tw] OR Vas Ligations [tw] OR Vas Occlusion* [tw] OR Intravasal Thread [tw] OR Intravasal Thread* [tw]	4,927
**21**	Intrauterine Devices [MeSH] OR Unmedicated IUDs [MeSH] OR Contraceptive Devices, Intrauterine [tw] OR Contraceptive IUD [tw] OR Contraceptive IUDs [tw] OR IUD, Unmedicated [tw] OR Unmedicated IUDs [tw] OR PPIUD, post-partum intrauterine device [tw] OR PPIUCD [tw] OR postpartum intrauterine contraceptive device [tw] OR Mirena [tw] OR libetta [tw] OR Kyleena [tw] OR skylab [tw] OR Jaydess [tw]	12,035
**19**	Levonorgestrel [MeSH] OR Microval [tw] OR Microlut [tw] OR Norgeston [tw] OR Norplant-2 [tw] OR NorLevo [tw] OR Norplant [tw] OR Plan B [tw] OR Vikela [tw] OR Cerazet [tw] OR Capronor [tw] OR Jadelle [tw] OR Implanon NXT [tw] OR Nexplanon [tw] OR Implanon [tw] OR Levoplant [tw]	5,156
**17**	Injections [MeSH] OR Injectables [MeSH] OR Medroxyprogesterone Acetate [MeSH] OR Norethindrone [MeSH] OR Subcutaneous injectables [tw] OR depot medroxyprogesterone acetate [tw] OR DMPA [tw] OR Subcutaneous DMPA [tw] OR Intramuscular DMPA [tw] OR Depo-Provera [tw] OR Farlutal [tw] OR Gestapuran [tw] OR Curretab [tw] OR Perlutex [tw] OR Provera [tw] OR Veramix [tw] OR Cycrin [tw] OR Medroxyprogesterone 17-Acetate, (6 alpha,17 alpha)-Isomer [tw] OR Conceplan [tw] OR Micronor [tw] OR Norlutin [tw] OR Nor-QD [tw]	1,473

	OR Norcolut [tw] OR Norcolute [tw] OR Norethindrone, (1 beta)-Isomer [tw] OR Monogest [tw] OR DMPA subQ in Uniject [tw] OR Depo [tw] OR Depo-Provera [tw] OR Sayana Press [tw] OR depo-subQ provera 104 [tw] OR Cyclo-Provera [tw] OR Lunella [tw] OR Lunelle [tw] OR Mesigyna [tw] OR Norigynon [tw]	
**15**	Contraceptives, Oral [MeSH] OR Contraceptives, Oral, Combined [MeSH] OR Oral Contraceptives [tw] OR Phasic Oral Contraceptives [tw] OR Low-Dose Contraceptives [tw] OR Oral, Combined Contraceptives [tw] OR Oral, Hormonal Contraceptives [tw] OR Oral, Sequential Contraceptives [tw] OR Oral, Synthetic Contraceptives [tw] OR postcoital [tw] OR Mifegyne [tw] OR Mifégyne [tw] OR Mifeprex [tw] OR l-Norgestrel [tw] OR l Norgestrel [tw] OR 18,19-dinorpregn-4-en-20-yn-3-one, 13-ethyl-17-hydroxy-, (17alpha)-(-)- D-Norgestrel [tw] OR D Norgestrel [tw] OR Microval [tw] OR Microlut [tw] OR Norgeston [tw] OR Norplant-2 [tw] OR Norplant 2 [tw] OR Norplant2 [tw] OR NorLevo [tw] OR Norplant [tw] OR Plan B [tw] OR Vikela [tw] OR Cerazet [tw]	1,603
**13**	General Practitioners [MeSH] OR Doctors [tw] OR Medical doctors [tw] OR Physicians [tw] OR Family doctors [tw] OR General practitioners [tw] OR Non specialist doctors [tw] OR Non specialist physicians [tw] OR Non specialist doctor*[tw]	458,16
**11**	Assistant medical officer [tw] OR Clinical officer [tw] OR Medical licentiate practitioner [tw] OR Health officer [tw] OR physician assistant [tw] OR surgical technician [tw] OR medical technician [tw] OR non-physician clinician [tw]	2,619
**10**	Clinical officer [tw] OR medical assistant [tw] OR health officer [tw] OR clinical associate [tw] OR non-physician clinician [tw]	10,429
**9**	Nurse Midwives [MeSH] OR Midwives [MeSH] OR Registered midwi* [tw] OR midwife [tw] OR community midwi*[tw] OR Midwives [tw] OR professional midwives [tw] OR midwifery [tw]	36,184
**8**	Nurses [MeSH] OR Registered Nurses [MeSH] OR Professional nurse [tw] OR Nurse practitioner [tw] OR Clinical nurse [tw] OR District nurse [tw] OR Public health nurse [tw] OR Nurse anesthetist [tw] OR Nurse educator [tw] OR Registered nurse [tw] OR clinical nurse specialist [tw] OR advanced practice nurse [tw] OR practice nurse [tw] OR licensed nurse [tw] OR diploma nurse [tw] OR BS nurse [tw] OR nurse clinician	102,599
**7**	Auxiliary midwife [MeSH] OR auxiliary nurse midwives [tw] OR auxiliary midwives [tw] OR midwife assistan* [tw] OR auxiliary nurse midwife [tw] OR auxiliary midwife [tw] OR midwifery assistan* [tw]	1,367
**5**	Allied Health Personnel [MeSH] OR Nursing Assistants [MeSH] OR Nurses' aides [MeSH] OR Nursing auxiliaries [tw] OR auxiliary nurses [tw] OR auxiliary nurse [tw] OR assistant nurses [tw] OR assistant nurse [tw] OR enrolled nurse* [tw] OR practical nurse* [tw] OR Healthcare Assistants [tw] OR Paramedics [tw] OR Population Program Specialists	57,912
**3**	Community Health Workers [MeSH] OR Traditional Birth Attendant [MeSH] OR Family planning personnel [tw] OR Village Health workers [tw] OR Barefoot Doctors [tw] OR Family planning personnel characteristics [tw] OR Community health extension workers [tw] OR community health aide* [tw] OR community health promoter* [tw] OR community volunteers [tw] OR voluntary health worker* [tw] OR Volunteers [tw] OR Traditional birth attendan* [tw] OR traditional healer* [tw] OR Village healers [tw] OR treatment supporters [tw] OR treatment promoter* [tw] OR Community Based Skilled Birth Attendant [tw] OR Dai [tw] OR Bidan Kampong [tw] OR Skilled Birth Attendants [tw] OR Dayas [tw] OR Traditional midwives [tw]	282,334
**2**	Self Care [MeSH] OR Self Administration [MeSH] OR Self Medication OR Auto-injection [tw] OR self-injection [tw] OR self-care [tw] OR self-administration [tw] OR self-medication	91,662

Embase (21st November 2020 and 5th February 2021)

('uterine tube sterilization'/exp/mj OR 'uterine tube sterilization' OR 'vasectomy'/exp/mj OR 'vasectomy' OR 'intrauterine contraceptive device'/exp/mj OR 'intrauterine contraceptive device' OR 'drug implant'/exp/mj OR 'drug implant' OR 'levonorgestrel'/exp/mj OR 'levonorgestrel' OR 'oral contraceptives'/exp/mj OR 'oral contraceptives' OR 'condom'/exp/mj OR 'condom' OR 'contraception'/exp/mj OR 'contraception' OR 'contraceptive agent'/exp/mj OR 'contraceptive agent' OR 'intramuscular drug administration'/exp OR 'intramuscular drug administration' OR 'subcutaneous drug administration'/exp/mj OR 'subcutaneous drug administration' OR (('uterine tube sterilization'/exp/mj OR 'uterine tube sterilization' OR 'vasectomy'/exp/mj OR 'vasectomy' OR 'intrauterine contraceptive device'/exp/mj OR 'intrauterine contraceptive device' OR 'drug implant'/exp/mj OR 'drug implant' OR 'levonorgestrel'/exp/mj OR 'levonorgestrel' OR 'oral contraceptives'/exp/mj OR 'oral contraceptives' OR 'condom'/exp/mj OR 'condom' OR 'contraception'/exp/mj OR 'contraception' OR 'contraceptive agent'/exp/mj OR 'contraceptive agent' OR 'intramuscular drug administration'/exp OR 'intramuscular drug administration' OR 'subcutaneous drug administration'/exp/mj OR 'subcutaneous drug administration') AND ('counseling'/exp/mj OR 'counseling'))) AND ('health auxiliary'/exp/mj OR 'health auxiliary' OR 'paramedical personnel'/exp/mj OR 'paramedical personnel' OR 'health care personnel'/exp/mj OR 'health care personnel' OR 'voluntary worker'/exp/mj OR 'voluntary worker' OR 'nurse'/exp/mj OR 'nurse' OR 'nursing assistant'/exp/mj OR 'nursing assistant' OR 'midwife'/exp/mj OR 'midwife' OR 'medical assistant'/exp/mj OR 'medical assistant' OR 'general practitioner'/exp/mj OR 'general practitioner' OR 'physician'/exp/mj OR 'physician' OR 'advanced practice provider'/exp/mj OR 'advanced practice provider' OR 'drug self administration'/exp/mj OR 'drug self administration') AND (2013:py OR 2014:py OR 2015:py OR 2016:py OR 2017:py OR 2018:py OR 2019:py OR 2020:py OR 2021:py)

Cochrane CENTRAL (1st December 2020)

ID Search
#1 [mh "Self Care"] OR [mh "Self Administration"] with Cochrane Library publication date Between Jan 2012 and Dec 2020
#2 [mh "Community Health Workers"] OR [mh "Traditional Birth Attendant"]
#3 [mh "Allied Health Personnel"] OR [mh "Nursing Assistants"] OR [mh "Nurses' aides"]
#4 [mh "Auxiliary midwife"] OR [mh "auxiliary nurse midwives"] OR [mh "auxiliary midwive"] OR [mh "midwife assistant"]
#5 [mh Nurses] OR [mh "Registered Nurses"] OR [mh "Professional nurse"] OR [mh "Nurse practitioner"] OR [mh " Clinical nurse "]
#6 [mh "Nurse Midwives"] OR [mh Midwives] OR [mh "Registered midwive"] OR [mh midwife]
#7 [mh "Clinical officer"] OR [mh "medical assistant"] OR [mh "health officer"] OR [mh "clinical associate"] OR [mh "non-physician clinician"]
#8 [mh "Assistant medical officer"] OR [mh "Clinical officer"]

#9 [mh "General Practitioners"] OR [mh "Doctors"] OR [mh "Medical doctors"]
#10 #1 OR #2 OR #3 OR #4 OR #5 OR #6 OR #7 OR #8 OR #9
#11 [mh Counseling] OR [mh "Contraceptive Effect"] OR [mh "Informed choice counseling"]
#12 [mh "Contraceptives, Oral"] OR [mh "Contraceptives, Oral, Combined"] OR [mh "Oral Contraceptives"]
#13 [mh "Injections"] OR [mh "Injectables"] OR [mh "injectable contraceptives "]
#14 [mh Levonorgestrel] OR [mh "implant contraceptives"]
#15 [mh IUD] OR [mh "Intrauterine Devices"] OR [mh "Unmedicated IUDs"] OR [mh "Contraceptive Devices, Intrauterine"] OR [mh "Contraceptive IUD"]
#16 [mh Vasectomy] OR [mh "Vas Ligation"] OR [mh "Vas Occlusion"] OR [mh "Vas Occlusion, Nonchemical Vasectomies"]
#17 [mh "Sterilization, Tubal"] OR [mh "Aldridge Procedure"]
#18 #11 AND (#12 OR #13 OR #14 OR #15 OR #16 OR #17)
#19 #18 OR #12 OR #13 OR #14 OR #15 OR #16 OR #17
#20 #10 AND #19 with Publication Year from 2012 to 2020, in Trials (Word variations have been searched)
