# Supplementary material for: Getting Up to Date with What Works: A Systematic Review on the Effectiveness and Safety of Task Sharing of Modern Methods in Family Planning Services
Source: Biomed Res Int. 2023 Feb 7;2023:8735563. doi: 10.1155/2023/8735563 (PMC9936454; doi:10.1155/2023/8735563)
Supplement: Supplementary 2 — Appendix B: review outcomes by contraceptive methods. [file 8735563.f2.docx]

**Table B.1: Overview of the review outcomes by contraceptive methods**

| Contraceptive methods | Outcome category | |
| --- | --- | --- |
|  | Safety | Effectiveness |
| Injectable contraceptives | Complications (injection sites reactions, needle stick injuries, post-procedure infections) | Continuation  Unintended pregnancies  Users’ satisfaction |
| Contraceptive implants | Complications (damage of vessels, tissues, infections, hematomas etc.) | Insertion failures  Continuation  Unintended pregnancies  Users’ satisfaction |
| IUDs/PPIUDs | Complications (uterine perforation, infections, pelvic pain, irregular bleeding etc.) | Insertion failures, expulsion,  removal  Continuation  Unintended pregnancies |
| Vasectomy /tubal ligation | Intraoperative and postoperative complications: bleedings, pain, infections, non-optimal wound healing etc. | Failure to complete procedure  Users’ satisfaction |
