# Supplementary material for: Getting Up to Date with What Works: A Systematic Review on the Effectiveness and Safety of Task Sharing of Modern Methods in Family Planning Services
Source: Biomed Res Int. 2023 Feb 7;2023:8735563. doi: 10.1155/2023/8735563 (PMC9936454; doi:10.1155/2023/8735563)
Supplement: Supplementary 3 — Appendix C: for PICO questions. [file 8735563.f3.docx]

**Table C.1: PICO Table**

| **P** | **I** | **C** | **O** |
| --- | --- | --- | --- |
| **Can lay health workers, pharmacists, pharmacy workers or women themselves deliver initiation of oral contraceptives as safely and effectively as the usual care?** | | | |
| Women of reproductive age | Initiation of oral contraceptives by lay health workers, pharmacists, pharmacy workers and women themselves | Initiation of oral contraceptives by usual care | Safety- serious side effects, infections, failure rates associated with injectables contraceptives  Coverage- initiation and continuation of oral contraceptive use  Satisfaction with provider |
| **Can lay health workers, auxiliary nurses, nurses, midwives, auxiliary nurse midwives, pharmacists, pharmacy workers and women themselves deliver injectable contraceptives as safely and effectively as the usual care?** | | | |
| Women of reproductive age | Delivery of injectable contraceptives by lay health workers, auxiliary nurses, nurses, midwives, auxiliary nurse midwives, pharmacists, pharmacy workers and women themselves | Delivery of injectable contraceptives by usual care (nurses, midwives, associate clinicians, doctors) | Safety- serious side effects, infections, failure rates associated with injectables contraceptives  Coverage- initiation and continuation of injectable contraceptive use  Satisfaction with provider |
| **Can lay health workers, auxiliary nurses, auxiliary nurse midwives, nurses, midwives, pharmacists and pharmacy workers provide insertion and removal of contraceptive implants as safely and effectively as the usual care?** | | | |
| Women of reproductive age | Delivery of contraceptive implants by lay health workers, auxiliary nurses, auxiliary nurse midwives, nurses, midwives, pharmacists and pharmacy workers | Delivery of contraceptive implants by usual care (associate clinicians and doctors) | Safety- serious side effects, infections, failure rates associated with contraceptive implants  Coverage- initiation or continuation of contraceptive implant use  Satisfaction with provider |
| **Can lay health workers, auxiliary nurses, auxiliary nurse midwives, nurses, midwives, pharmacists and pharmacy workers provide insertion and removal of IUDs as safely and effectively as the usual care?** | | | |
| Women of reproductive age | Insertion and removal of IUDs by lay health workers lay health workers, auxiliary nurses, auxiliary nurse midwives, nurses, midwives, pharmacists and pharmacy workers | Insertion and removal of IUDs by usual care (associate clinicians and doctors) | Safety- serious side effects, infections, failure rates associated with IUDs  Coverage- initiation or continuation of IUDs use  Satisfaction with provider |
| **Can nurses, midwives, auxiliary nurses, auxiliary nurse midwives perform vasectomy as safely and effectively as the usual care?** | | | |
| Men of reproductive age | Nurses, midwives, auxiliary nurses, auxiliary nurse midwives performing vasectomy | Usual care (associate clinicians and doctors) performing vasectomy | Safety- serious side effects, infections, failure rates associated with vasectomy  Coverage- initiation of vasectomy use   Satisfaction with provider |
| **Can nurses and midwives perform tubal ligation as safely and effectively as the usual care?** | | | |
| Women of reproductive age | Nurses and midwives performing tubal ligation | Usual care (associate clinicians and doctors) performing tubal ligation | Safety- serious side effects, infections, failure rates associated with tubal ligation  Coverage- initiation of tubal ligation use  Satisfaction with provider |
